# Social prescribing in Canada: linking the Ottawa Charter for Health Promotion with health care’s Quintuple Aim for a collaborative approach to health

**DOI:** 10.24095/hpcdp.44.9.01

**Published:** 2024-09

**Authors:** Kate Mulligan, Kiffer G. Card, Sandra Allison

**Affiliations:** 1 Dalla Lana School of Public Health, University of Toronto, Toronto, Ontario, Canada; 2 Faculty of Health Sciences, Simon Fraser University, Vancouver, British Columbia, Canada; 3 University of British Columbia, Vancouver, British Columbia, Canada

**Keywords:** social prescribing, Ottawa Charter for Health Promotion, Quintuple Aim for health care improvement

## Abstract

Social prescribing offers a practical mechanism by which public health and health care systems can work together toward a future in which well-being is prioritized, health equity is addressed and people and communities thrive. The articles in this second part of the *Health Promotion and Chronic Disease Prevention in Canada* special issue on social prescribing explore how social prescribing in Canada supports action on two frameworks important to public health and health care communities: the Ottawa Charter for Health Promotion, which emphasizes building healthy public policy, creating supportive environments, strengthening community action, developing personal skills and reorienting health services, and the Quintuple Aim for health care improvement, which focusses on improved population health, health equity, patient experience, care team well-being and reduced costs.

**Highlights**

- Social prescribing supports collaboration between public health and health care services by providing a mechanism for action on both the Ottawa Charter for Health Promotion and the Quintuple Aim for health care improvement.
- At the individual level, people develop personal skills (Ottawa Charter), and care experiences improve for participants, patients and health care workers (Quintuple Aim).
- At the community level, health service reorientation strengthens community action, builds supportive environments and reduces acute care costs by moving care upstream.
- At the population level, precision data on health and social care support prioritization and decision making for healthy public policy and health equity.

## Introduction

Social prescribing continues to grow rapidly across Canada, complementing existing strengths and building capacity for improving how we address health promotion and chronic disease prevention in Canada. The first part of this special issue of *Health Promotion and Chronic Disease Prevention in Canada* (HPCDP) on social prescribing (published in June 2024) described the practice of social prescribing across settings, populations and interventions, with a focus on the role of communities and community organizations. 

This second part speaks primarily to public health and health care communities, who are respectively guided by two crucial frameworks: the Ottawa Charter for Health Promotion^1^ and the Quintuple Aim for health care improvement.^2^ The Ottawa Charter, established by the World Health Organization in 1986, describes health promotion as a process of empowering people and communities to take more control over their health and its determinants. The Charter outlines five action areas for health promotion: building healthy public policy, creating supportive environments, strengthening community action, developing personal skills and reorienting health services. The Quintuple Aim, developed by the Institute for Healthcare Improvement, expands on the traditional triple aim for better health care (patient or participant experience, population health and reduced costs) by adding clinician or care team well-being and addressing health equity. The articles in this second part of our special social prescribing issue explore how social prescribing research, policies and practices in Canada align with these frameworks, as outlined here.

## Develop personal skills (Ottawa Charter) and improve patient experience (Quintuple Aim)

Social prescribing is a strengths-based approach that supports people in exercising and developing personal skills, such as financial literacy, cooking skills, advocacy or leadership, that support self-determination—a health promotion approach rooted in individual and collective autonomy, competence, relatedness and beneficence.^3^ These skills vary across populations, geographies and the life course, as demonstrated by Yu et al.’s qualitative analysis of the expressed social prescribing needs and priorities of older adults.^4 ^ Increasingly, research shows that relationships built with social prescribing link workers—often peers from a shared community—are important to this skill development and are correlated with an improvement in experience.^5^ The connection to a supportive community health worker helps to support and sustain people and distinguishes social prescribing from a less personalized approach focussed first and foremost on care or service navigation; link workers not only provide social referrals, but befriend participants and bear witness to their distress.^5^

## Strengthen community action (Ottawa Charter) and address health equity (Quintuple Aim)

Social prescribing programs can help bridge the gap in health outcomes experienced by different populations, both by supporting communities in identifying and addressing their own health needs and by connecting patients with resources they may not have otherwise accessed.^6^ Part I of our special issue on social prescribing includes examples of Afrocentric^7^ and reconciliation-based^8^ community development in social prescribing. In this issue, Kadowaki et al.’s mixed methods analysis demonstrates how social prescribing in British Columbia improved access to services for older adults, but also established a clear need for stronger and more stable resources for existing and new community programs.^9^

## Reorient health services (Ottawa Charter) and reduce costs (Quintuple Aim)

Social prescribing provides a mechanism for meaningful collaboration between health care and community organizations on addressing health-related social needs.^10^ It also demonstrably supports deprescribing, allows for more efficient upstream health spending by moving care upstream and reduces health care’s environmental impacts by preventing unnecessary health care utilization.^11^ Saluja and Dahrouge’s contribution from the Access to Resources in the Community project in Ottawa provides detailed guidance for how to reorient services within health care settings,^12^ while Lin and colleagues’ commentary from BC’s Fraser Health Authority demonstrates the value of long-term funding and strong support from within health care organizations to initiate and sustain community-partnered social prescribing.^13^

## Create supportive environments (Ottawa Charter) and improve care team well-being (Quintuple Aim)

Social prescribing creates and supports connections to, and resources for, healthy social and physical places, such as community gardens and cultural centres, that foster a sense of belonging, social interaction and a connection to nature.^14^ This impact extends to the well-being of strained health human resources, offering a sense of connection, purpose and belonging for clinicians.^15^ The quantitative study by Turpin et al. of Youth Wellness Hubs in Ontario demonstrates the benefits of a service hub approach,^16^ whereby multiple youth wellness services are most often provided in a single, community space, for coordination among multidisciplinary care teams and across clinical and nonclinical services.

## Build healthy public policy (Ottawa Charter) and improve population health outcomes (Quintuple Aim)

Social prescribing can improve population health at scale by promoting healthy behaviours, social connections and access to community resources for all users of health and social services.^17^ Crucially, it can inform healthy public policy priorities and decisions through precision data collection that helps to identify the community resources that participants need most.^18^ Most social prescribing initiatives are well connected to policy development in their respective regions, as demonstrated by Mansell et al.’s policy brief^19^ linking social prescribing evaluation and policy as guided by the Healthy Aging Asset Index in Alberta.

## Conclusion

Social prescribing represents a significant step forward in achieving the goals outlined in both the Ottawa Charter and the Quintuple Aim. It fosters a holistic approach to health care, recognizing the interconnectedness of social, environmental and individual factors that influence health. By investing in social prescribing programs, public health and health care systems can move towards a future in which well-being is prioritized, health equity is addressed and both communities and health and social care workers thrive. As we strive for a healthier Canada, social prescribing offers a powerful and practical tool to navigate the path forward.

## Conflicts of interest

KM, KGC, and SA were Guest Editors for this issue of the HPCDP Journal, but removed themselves from the editorial decision-making associated with this manuscript.

## Statement

The content and views expressed in this article are those of the authors and do not necessarily reflect those of the Government of Canada.
